# *N*-(2,3,4-Trifluoro­phen­yl)morpholine-4-carboxamide

**DOI:** 10.1107/S1600536811051300

**Published:** 2011-12-03

**Authors:** Li Jie, Pei Shuchen, Hai Li, Wu Yong

**Affiliations:** a Key Laboratory of Drug Targeting of the Education Ministry, West China School of Pharmacy, Sichuan University, Chengdu 610041, People’s Republic of China

## Abstract

In title mol­ecule, C_11_H_11_F_3_N_2_O_2_, the central –N—C(=O)—N– unit is essentially planar [maximum deviation = 0.013 (2) Å] and forms a dihedral angle of 57.33 (9)° with the benzene ring. The morpholine ring is in a chair conformation. In the crystal, mol­ecules are linked into chains along [001] by N—H⋯O hydrogen bonds.

## Related literature

For background to urea derivatives as anti­bacterial and anti­fungal agents, see: Zheng *et al.* (2010 ▶).
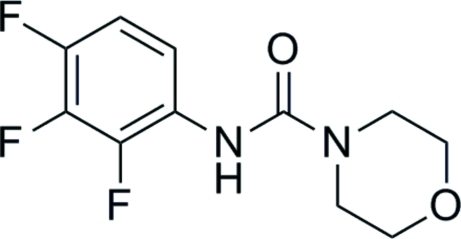


## Experimental

### 

#### Crystal data

C_11_H_11_F_3_N_2_O_2_
*M* *_r_* = 260.22
Monoclinic, 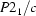

*a* = 7.8515 (4) Å
*b* = 17.8264 (6) Å
*c* = 8.6872 (4) Å
β = 109.790 (5)°
*V* = 1144.09 (8) Å^3^
*Z* = 4
Mo *K*α radiation
μ = 0.14 mm^−1^
*T* = 293 K
0.40 × 0.35 × 0.30 mm

#### Data collection

Agilent Xcalibur Eos diffractometer
Absorption correction: multi-scan (*CrysAlis PRO*; Agilent, 2011 ▶) *T*_min_ = 0.994, *T*_max_ = 1.000
4250 measured reflections
2009 independent reflections
1587 reflections with *I* > 2σ(*I*)
*R*_int_ = 0.016

#### Refinement

*R*[*F* ^2^ > 2σ(*F* ^2^)] = 0.041
*wR*(*F* ^2^) = 0.103
*S* = 1.07
2009 reflections
167 parameters
H atoms treated by a mixture of independent and constrained refinement
Δρ_max_ = 0.15 e Å^−3^
Δρ_min_ = −0.23 e Å^−3^

### 

Data collection: *CrysAlis PRO* (Agilent, 2011) ▶; cell refinement: *CrysAlis PRO* ▶; data reduction: *CrysAlis PRO* ▶; program(s) used to solve structure: *SHELXS97* (Sheldrick, 2008 ▶); program(s) used to refine structure: *SHELXL97* (Sheldrick, 2008 ▶); molecular graphics: *OLEX2* (Dolomanov *et al.*, 2009 ▶) and *PLATON* (Spek, 2009 ▶); software used to prepare material for publication: *OLEX2*.

## Supplementary Material

Crystal structure: contains datablock(s) global, I. DOI: 10.1107/S1600536811051300/lh5385sup1.cif

Structure factors: contains datablock(s) I. DOI: 10.1107/S1600536811051300/lh5385Isup2.hkl

Supplementary material file. DOI: 10.1107/S1600536811051300/lh5385Isup3.cdx

Supplementary material file. DOI: 10.1107/S1600536811051300/lh5385Isup4.cml

Additional supplementary materials:  crystallographic information; 3D view; checkCIF report

## Figures and Tables

**Table 1:** Hydrogen-bond geometry (Å, °)

*D*—H⋯*A*	*D*—H	H⋯*A*	*D*⋯*A*	*D*—H⋯*A*
N1—H1⋯O2^i^	0.843 (18)	2.120 (19)	2.9306 (19)	161.2 (17)

Symmetry code: (i) 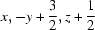.

**Fig. 1.**
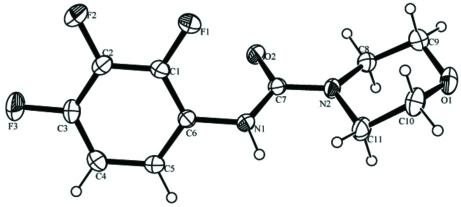
The molecular structure of the title compound, with displacement ellipsoids drawn at the 50% probability level.

**Fig. 2.**
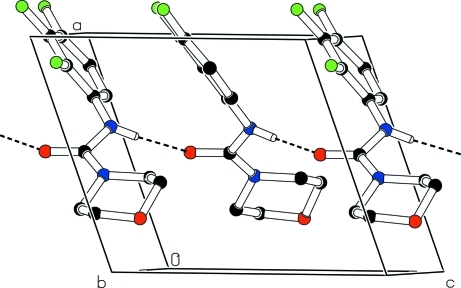
Part of the crystal structure with hydrogen bonds shown as dashed lines.

## supplementary crystallographic information

### Comment

The title compound (I) was made as part of our work on urea derivatives. Urea derivatives have been reported in the literature as antibacterial and antifungal agents (Zheng *et al.*, 2010). The crystal structure of (I) is reported herein here.

The molecular structure of the title compound is shown in Fig. 1. The central -N—C(═O)—N- unit is essentially planar (the maximun deviation from atoms N2/C7/O2/O1 is 0.013Å for C7) and forms a dihedral angle of 57.33 (9)° with the benzene ring. The morpholine ring is in a chair conformation. In the crystal, molecules are linked into chains along [001] by N—H···O hydrogen bonds (Fig. 2).

### Experimental

To a solution of triphosgene (350 mg, 1.19 mmol) and triethylamine (680 mg, 6.80 mmol) in anhydrous acetonitrile (5 ml) at ice bath, a solution of 2,3,4-trifluoroaniline (500 mg, 3.40 mmol) and triethylamine (680 mg, 6.80 mmol) in anhydrous acetonitrile (5 ml) was added dropwise. The mixture was stirred for 1 h. Then, a solition of morpholine (300 mg,3.40 mmol) and triethylamine (680 mg, 6.80 mmol) in anhydrous acetonitrile (5 ml) was added dropwise. The reaction mixture was then removed from the cooling bath and stirred at room temperature overnight. On completion of the reaction, the mixture was poured into water. The aqueous layer was extracted with ethyl acetate and the organic layer was separated. The organic layers were washed with brine and dried over sodium sulfate, filtered, and concentrated *in vacuo*. The purification of the residue by silica gel column chromatography eluting with EtOAc-petroleum ether (1:8) yielded the white solid 670 mg (yield 76.2%) of *N*-(2,3,4-trifluorophenyl)morpholine-4-carboxamide. Colorless crystals suitable for X-ray analysis were obtained by slow evaporation in ethyl acetate at room temperature.

### Refinement

H atoms bonded to C atoms were positioned geometrically (C—H = 0.93–0.97 Å) and refined using a riding-model approximation, with *U*_iso_(H) = 1.2*U*_eq_(C). The H atom bonded to N was refined independently with an isotropic displacement parameter.

### Crystal data

**Table:**

C_11_H_11_F_3_N_2_O_2_	*F*(000) = 536
*M**_r_* = 260.22	*D*_x_ = 1.511 Mg m^−^^3^
Monoclinic, *P*2_1_/*c*	Mo *K*α radiation, λ = 0.7107 Å
*a* = 7.8515 (4) Å	Cell parameters from 1772 reflections
*b* = 17.8264 (6) Å	θ = 3.0–29.1°
*c* = 8.6872 (4) Å	µ = 0.14 mm^−^^1^
β = 109.790 (5)°	*T* = 293 K
*V* = 1144.09 (8) Å^3^	Block, colorless
*Z* = 4	0.40 × 0.35 × 0.30 mm

### Data collection

**Table:**

Agilent Xcalibur Eos diffractometer	2009 independent reflections
Radiation source: Enhance (Mo) X-ray Source	1587 reflections with *I* > 2σ(*I*)
graphite	*R*_int_ = 0.016
Detector resolution: 16.0874 pixels mm^-1^	θ_max_ = 25.0°, θ_min_ = 3.0°
ω scans	*h* = −7→9
Absorption correction: multi-scan (*CrysAlis PRO*; Agilent, 2011)	*k* = −13→21
*T*_min_ = 0.994, *T*_max_ = 1.000	*l* = −10→7
4250 measured reflections	

### Refinement

**Table:**

Refinement on *F*^2^	Primary atom site location: structure-invariant direct methods
Least-squares matrix: full	Secondary atom site location: difference Fourier map
*R*[*F*^2^ > 2σ(*F*^2^)] = 0.041	Hydrogen site location: inferred from neighbouring sites
*wR*(*F*^2^) = 0.103	H atoms treated by a mixture of independent and constrained refinement
*S* = 1.07	*w* = 1/[σ^2^(*F*_o_^2^) + (0.044*P*)^2^ + 0.1807*P*] where *P* = (*F*_o_^2^ + 2*F*_c_^2^)/3
2009 reflections	(Δ/σ)_max_ < 0.001
167 parameters	Δρ_max_ = 0.15 e Å^−^^3^
0 restraints	Δρ_min_ = −0.23 e Å^−^^3^

### Special details

**Table:**

Geometry. All e.s.d.'s (except the e.s.d. in the dihedral angle between two l.s. planes) are estimated using the full covariance matrix. The cell e.s.d.'s are taken into account individually in the estimation of e.s.d.'s in distances, angles and torsion angles; correlations between e.s.d.'s in cell parameters are only used when they are defined by crystal symmetry. An approximate (isotropic) treatment of cell e.s.d.'s is used for estimating e.s.d.'s involving l.s. planes.
Refinement. Refinement of *F*^2^ against ALL reflections. The weighted *R*-factor *wR* and goodness of fit *S* are based on *F*^2^, conventional *R*-factors *R* are based on *F*, with *F* set to zero for negative *F*^2^. The threshold expression of *F*^2^ > σ(*F*^2^) is used only for calculating *R*-factors(gt) *etc*. and is not relevant to the choice of reflections for refinement. *R*-factors based on *F*^2^ are statistically about twice as large as those based on *F*, and *R*- factors based on ALL data will be even larger.

### Fractional atomic coordinates and isotropic or equivalent isotropic displacement parameters (Å^2^)

**Table:**

	*x*	*y*	*z*	*U*_iso_*/*U*_eq_	
F1	0.88764 (17)	0.67437 (6)	0.52547 (15)	0.0574 (4)	
F2	1.12312 (17)	0.76456 (7)	0.45200 (16)	0.0681 (4)	
F3	1.11974 (18)	0.91342 (7)	0.50200 (18)	0.0764 (4)	
O1	0.2304 (2)	0.51651 (8)	0.66738 (18)	0.0617 (4)	
O2	0.50780 (18)	0.68346 (7)	0.37155 (14)	0.0427 (4)	
N1	0.6148 (2)	0.73140 (8)	0.63043 (18)	0.0384 (4)	
N2	0.4106 (2)	0.63276 (8)	0.56564 (16)	0.0370 (4)	
C1	0.8741 (3)	0.74877 (10)	0.5421 (2)	0.0380 (4)	
C2	0.9976 (3)	0.79446 (11)	0.5078 (2)	0.0434 (5)	
C3	0.9947 (3)	0.87006 (11)	0.5337 (2)	0.0469 (5)	
C4	0.8722 (3)	0.90070 (11)	0.5949 (2)	0.0471 (5)	
H4	0.8739	0.9519	0.6159	0.057*	
C5	0.7451 (3)	0.85485 (10)	0.6255 (2)	0.0397 (5)	
H5	0.6604	0.8757	0.6662	0.048*	
C6	0.7420 (2)	0.77841 (9)	0.59651 (19)	0.0331 (4)	
C7	0.5081 (2)	0.68272 (9)	0.5135 (2)	0.0328 (4)	
C8	0.2658 (3)	0.59059 (10)	0.4469 (2)	0.0422 (5)	
H8A	0.2909	0.5865	0.3452	0.051*	
H8B	0.1523	0.6173	0.4244	0.051*	
C9	0.2489 (3)	0.51341 (11)	0.5100 (3)	0.0546 (6)	
H9A	0.1442	0.4885	0.4342	0.065*	
H9B	0.3553	0.4842	0.5165	0.065*	
C10	0.3837 (3)	0.55182 (12)	0.7792 (2)	0.0587 (6)	
H10A	0.4916	0.5240	0.7841	0.070*	
H10B	0.3735	0.5512	0.8874	0.070*	
C11	0.4023 (3)	0.63175 (11)	0.7304 (2)	0.0484 (5)	
H11A	0.2996	0.6610	0.7340	0.058*	
H11B	0.5115	0.6538	0.8063	0.058*	
H1	0.576 (3)	0.7459 (10)	0.705 (2)	0.039 (5)*	

### Atomic displacement parameters (Å^2^)

**Table:**

	*U*^11^	*U*^22^	*U*^33^	*U*^12^	*U*^13^	*U*^23^
F1	0.0633 (8)	0.0337 (6)	0.0872 (9)	0.0021 (5)	0.0412 (7)	−0.0068 (6)
F2	0.0613 (8)	0.0643 (8)	0.1001 (10)	0.0016 (6)	0.0552 (8)	−0.0071 (7)
F3	0.0789 (10)	0.0583 (8)	0.1138 (11)	−0.0191 (7)	0.0613 (9)	0.0059 (7)
O1	0.0655 (10)	0.0587 (10)	0.0722 (10)	−0.0197 (8)	0.0380 (9)	0.0057 (8)
O2	0.0571 (9)	0.0439 (8)	0.0345 (7)	−0.0081 (6)	0.0253 (6)	−0.0018 (5)
N1	0.0493 (10)	0.0379 (9)	0.0361 (8)	−0.0088 (7)	0.0252 (8)	−0.0071 (7)
N2	0.0437 (9)	0.0384 (8)	0.0333 (8)	−0.0090 (7)	0.0188 (7)	0.0005 (6)
C1	0.0448 (11)	0.0291 (9)	0.0438 (10)	0.0012 (8)	0.0199 (9)	−0.0017 (8)
C2	0.0428 (11)	0.0460 (12)	0.0497 (11)	0.0019 (9)	0.0266 (9)	−0.0013 (9)
C3	0.0494 (12)	0.0427 (11)	0.0549 (12)	−0.0112 (10)	0.0259 (10)	0.0050 (9)
C4	0.0572 (13)	0.0308 (10)	0.0576 (12)	−0.0045 (9)	0.0252 (11)	−0.0012 (9)
C5	0.0449 (11)	0.0349 (10)	0.0435 (10)	0.0010 (9)	0.0206 (9)	−0.0045 (8)
C6	0.0398 (10)	0.0326 (9)	0.0294 (8)	−0.0031 (8)	0.0150 (8)	−0.0008 (7)
C7	0.0379 (10)	0.0300 (9)	0.0338 (9)	0.0033 (8)	0.0165 (8)	0.0014 (7)
C8	0.0408 (11)	0.0433 (11)	0.0449 (11)	−0.0065 (9)	0.0177 (9)	−0.0050 (9)
C9	0.0563 (14)	0.0431 (12)	0.0725 (14)	−0.0106 (10)	0.0326 (12)	−0.0082 (10)
C10	0.0637 (15)	0.0638 (14)	0.0549 (13)	−0.0065 (12)	0.0283 (12)	0.0179 (11)
C11	0.0588 (13)	0.0536 (12)	0.0389 (10)	−0.0124 (10)	0.0247 (9)	0.0023 (9)

### Geometric parameters (Å, °)

**Table:**

F1—C1	1.342 (2)	C3—C4	1.361 (3)
F2—C2	1.347 (2)	C4—H4	0.9300
F3—C3	1.349 (2)	C4—C5	1.383 (3)
O1—C9	1.424 (2)	C5—H5	0.9300
O1—C10	1.413 (3)	C5—C6	1.384 (2)
O2—C7	1.232 (2)	C8—H8A	0.9700
N1—C6	1.409 (2)	C8—H8B	0.9700
N1—C7	1.383 (2)	C8—C9	1.504 (3)
N1—H1	0.843 (18)	C9—H9A	0.9700
N2—C7	1.349 (2)	C9—H9B	0.9700
N2—C8	1.459 (2)	C10—H10A	0.9700
N2—C11	1.455 (2)	C10—H10B	0.9700
C1—C2	1.374 (3)	C10—C11	1.508 (3)
C1—C6	1.382 (2)	C11—H11A	0.9700
C2—C3	1.368 (3)	C11—H11B	0.9700
			
C10—O1—C9	109.68 (15)	O2—C7—N2	122.31 (16)
C6—N1—H1	116.2 (13)	N2—C7—N1	116.05 (15)
C7—N1—C6	121.05 (14)	N2—C8—H8A	109.5
C7—N1—H1	118.2 (13)	N2—C8—H8B	109.5
C7—N2—C8	119.83 (14)	N2—C8—C9	110.94 (15)
C7—N2—C11	123.95 (15)	H8A—C8—H8B	108.0
C11—N2—C8	113.93 (15)	C9—C8—H8A	109.5
F1—C1—C2	118.37 (17)	C9—C8—H8B	109.5
F1—C1—C6	120.70 (16)	O1—C9—C8	111.42 (17)
C2—C1—C6	120.91 (17)	O1—C9—H9A	109.3
F2—C2—C1	119.96 (17)	O1—C9—H9B	109.3
F2—C2—C3	120.24 (18)	C8—C9—H9A	109.3
C3—C2—C1	119.78 (18)	C8—C9—H9B	109.3
F3—C3—C2	118.52 (18)	H9A—C9—H9B	108.0
F3—C3—C4	120.61 (18)	O1—C10—H10A	109.3
C4—C3—C2	120.83 (18)	O1—C10—H10B	109.3
C3—C4—H4	120.4	O1—C10—C11	111.65 (17)
C3—C4—C5	119.28 (18)	H10A—C10—H10B	108.0
C5—C4—H4	120.4	C11—C10—H10A	109.3
C4—C5—H5	119.5	C11—C10—H10B	109.3
C4—C5—C6	121.08 (18)	N2—C11—C10	109.20 (16)
C6—C5—H5	119.5	N2—C11—H11A	109.8
C1—C6—N1	120.74 (15)	N2—C11—H11B	109.8
C1—C6—C5	117.99 (17)	C10—C11—H11A	109.8
C5—C6—N1	121.17 (16)	C10—C11—H11B	109.8
O2—C7—N1	121.59 (16)	H11A—C11—H11B	108.3

### Hydrogen-bond geometry (Å, °)

**Table:**

*D*—H···*A*	*D*—H	H···*A*	*D*···*A*	*D*—H···*A*
N1—H1···O2^i^	0.843 (18)	2.120 (19)	2.9306 (19)	161.2 (17)

Symmetry codes: (i) *x*, −*y*+3/2, *z*+1/2.
